# Intrinsic over extrinsic: Species identity shapes spatial and interannual Mg/Ca patterns in Arctic marine calcifiers

**DOI:** 10.1371/journal.pone.0345703

**Published:** 2026-03-20

**Authors:** Małgorzata Krzemińska, Emma Humphreys-Williams, Tomasz Krzykawski, Piotr Kukliński

**Affiliations:** 1 Institute of Oceanology, Polish Academy of Sciences, Sopot, Poland; 2 University College of London, Department of Earth Sciences, London, United Kingdom; 3 University of Silesia, Faculty of Earth Sciences, Sosnowiec, Poland; King Abdulaziz University, SAUDI ARABIA

## Abstract

Marine calcifiers incorporate magnesium into their calcium carbonate skeletons through processes influenced by both ambient environmental conditions and species-specific physiological regulation. As a result, their carbonate structures can serve as valuable archives of past and present oceanic conditions, provided that biological controls are explicitly considered. This study investigated how skeletal magnesium-to-calcium (Mg/Ca) ratios vary in space and time among three Arctic benthic invertebrates differing in phylogeny, evolutionary history, and biomineralization strategy: the barnacle *Semibalanus balanoides*, the spirorbid *Paradexiospira violacea*, and the bryozoan *Harmeria scutulata*. Mg/Ca ratios were quantified using inductively coupled plasma atomic emission spectroscopy (ICP-AES) across three Svalbard fjords and over a four-year temporal interval (2006–2009). Kruskal–Wallis analyses revealed that species identity was the dominant factor controlling Mg/Ca ratios among the studied taxa. *S. balanoides* exhibited the lowest values of mean Mg/Ca ratios but highly variable (mean = 35.2 mmol/mol ± 16.8 SD), whereas *P. violacea* (62.5 mmol/mol ± 14.3) and *H. scutulata* (65.3 mmol/mol ± 14.1) showed higher and more consistent Mg/Ca ratio. Significant differences in Mg/Ca ratios were observed among sites for all species, following a consistent Hornsund < Kongsfjorden < Isfjorden pattern. However, the magnitude of site-level variability in Mg/Ca differed among species, confirming that species-specific physiological controls exert a stronger influence on skeletal Mg incorporation than external, site-specific environmental conditions. In Isfjorden, interannual trends in Mg/Ca between 2006 and 2009 were found to be species-specific but non-significant for all species. Weak, negative, and statistically significant relationships with bottom-water temperatures in Isfjorden was found only in *P. violacea.* Overall, these results highlight the predominant role of intrinsic biological controls over ambient environmental conditions in shaping skeletal Mg/Ca ratios and underscore the importance of species-resolved approaches when applying geochemical proxies in rapidly changing Arctic ecosystems.

## Introduction

Calcium carbonate (CaCO₃) biomineralization is a key evolutionary innovation that has shaped the history of life and the global carbon cycle for over 540 million years [1]. Since the Cambrian and Ordovician radiations, the production of skeletal carbonate by animals and coralline algae serve not only as protection in the form of exoskeletons or structural support but also as sink for carbon, playing an important role in the regulation of atmospheric CO₂ over geological timescales [2,3]

The primary polymorphs of calcium carbonate used by marine invertebrates for skeletal construction are calcite and aragonite, which can be precipitated either independently or combined depending on the organism and environmental conditions [4]. Biogenic calcite, typically very pure (~99% CaCO₃), incorporates a suite of elements among which magnesium (Mg) is the most common impurity and plays a critical role in modifying its structural and geochemical properties. As Mg² ⁺ substitutes for Ca²⁺ in the calcite crystal lattice, it increases internal compressive stress due to its smaller ionic radius. This has functional significance for invertebrates resulting in enhanced mechanical hardness and resistance to fracture and, improved skeletal durability in dynamic environments [5]. Beyond its structural role, magnesium incorporation into calcite is widely used as a geochemical proxy, most notably as a paleotemperature indicator, because it increases with ambient temperature during calcification [6]. This relationship has been validated in numerous marine taxa, such as foraminifera and ostracods, and forms the basis for reconstructing historical ocean temperatures. In addition to temperature, other environmental variables such as e.g. pH or carbonate saturation state (Ω) influence magnesium incorporation [7,8].

Th environmental signal recorded in biogenic calcite, however can be modified by species-specific physiological and metabolic processes, collectively known as “vital effects”, which may obscure or alter environmental information [7,9]. These effects include biological regulation of the calcifying fluid’s chemistry, such as control over pH, ion concentration, and dissolved inorganic carbon [9]. Consequently, any interpretation of skeletal geochemistry must consider the contribution of biological traits alongside environmental drivers.

This study aim to investigate the relationship between skeletal geochemistry, environmental variability in sessile marine invertebrates across Svalbard. Focusing on three different taxa (polychaetes, barnacles, and bryozoans), the major question is whether skeletal Mg-calcite distribution is species-specific or controlled with environmental conditions associated with various spatial (site, fjord, depth) and temporal scales (four years, between 2006 and 2009). Understanding how polar marine calcifiers respond to ongoing climate change is essential for predicting the resilience and vulnerability of calcifying communities under increasing environmental stress, particularly in the rapidly changing Arctic. To address study objective, we tested three specific hypotheses: 1. Each species will display unique patterns in Mg/Ca distribution, reflecting intrinsic physiological and biomineralization differences; 2. Skeletal Mg/Ca ratios will vary with local environmental conditions such as, e.g., temperature, salinity, and carbonate chemistry, but these responses will be species-specific; 3. Interannual variation in skeletal Mg/Ca ratios will correspond to temporal fluctuations in seawater temperature.

## Materials and methods

### Study site and sampling protocol

**Fig 1:**
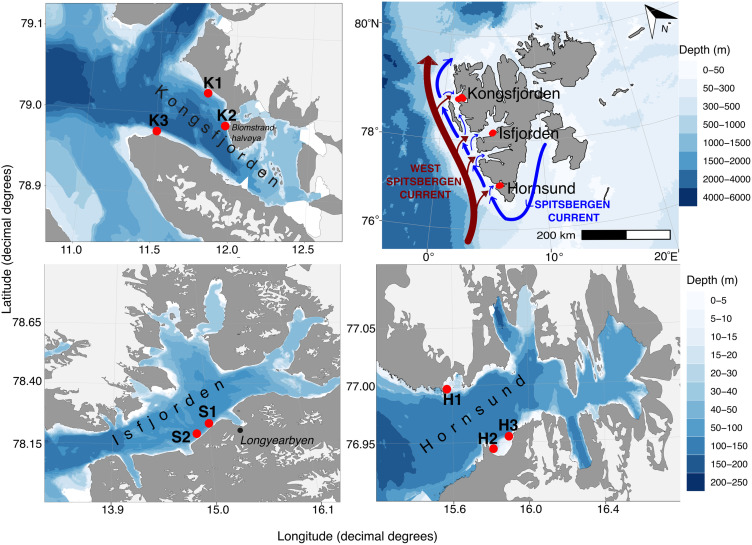
Study area with sampling locations (red dots) distributed in three Svalbard fjords: Kongsfjorden, Isfjorden and Hornsund. Maps were created using the *PlotSvalbard* R package [10] distributed under the CC BY 4.0 license [10].

Svalbard fjords such as Isfjorden, Kongsfjorden, and Hornsund (Fig 1) are high-Arctic systems shaped by the interplay of warm, saline Atlantic Water (AW) from the West Spitsbergen Current and cold, fresh Arctic Water (ArW) from the Coastal Current [11–23]. Hornsund represents the coldest fjord system, with surface temperatures below ~3 °C and comparatively low salinities (<31 close to the glaciers 33–34 in central fjord), driven primarily by glacial meltwater input [11–20,23]. The fjord receives substantial nutrient enrichment from seabird colonies, supporting high turbidity and primary production (~216 g C m ⁻ ² y ⁻ ¹) dominated by microplankton, indicative of strong Arctic nutrient availability [15,16]. Isfjorden exhibits surface temperatures around 4.9 °C and salinities exceeding 34.7 PSU, reflecting a mixture of Arctic and Atlantic water masses [21]. Kongsfjorden is the warmest fjord, approximately 1 °C warmer than Hornsund and about 0.5 PSU more saline. However, it experiences episodic surface freshening below 28 PSU, lower primary production ~48 g C m ⁻ ² y ⁻ ¹ [18,22,23] and reduced calcite saturation relative to Hornsund [17,22–24].

The study was conducted around Spitsbergen Island, Svalbard, across three fjords: Kongsfjorden (K1: 79°01.8′ N, 11°49.8′ E; K2: 78°59.5′ N, 11°58.9′ E; K3: 78°58.5′ N, 11°29.8′ E), Isfjorden (S1: 78°12.7′ N, 15°14.1′ E; S2: 78°11.2′ N, 15°08.7′ E), and Hornsund (H1: 77°00.8′ N, 15°33.3′ E; H2: 76°56.8′ N, 15°48.4′ E; H3: 76°57.4′ N, 15°55.6′ E; Fig 1, S1 Appendix). Access to the field sites and permission to conduct sampling were granted by the Governor of Svalbard (Sysselmannen på Svalbard; ref: 2007/00652–2, a.512).

Sampling covered both local (<10 m) and regional (>250 km) spatial scales. Divers collected specimens at standardized depths (6 and 12 m). In Kongsfjorden and Hornsund, ≥ 25 boulders per site were sampled at intervals >10 m, with organisms spaced ≥10 cm apart. In Isfjorden, three 15 × 15 cm experimental panels were deployed at S1 and S2 (2005–2009) and replaced annually [25]. Temperatures were recorded from 2006 to 2009 using HOBO loggers (U22-001, UA-002–64) attached to panel frames. Sea temperatures at collection sites averaged ~3 °C, ranging annually from −1.8 °C to +4 °C.

**Fig 2:**
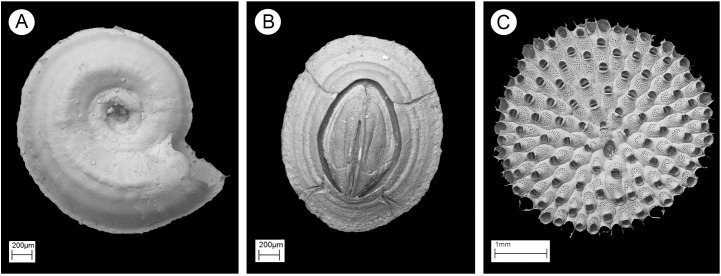
SEM images by PK, of targeted taxa. *Paradexiospira violacea* (a); *Semibalanus balanoides* (b); *Harmeria scutulata* c).

This study examines three widespread marine invertebrates (Fig 2): the Arctic spirorbid polychaete *Paradexiospira violacea* (Levinsen)*,* the circumpolar bryozoan *Harmeria scutulata* (Busk) and the boreo-temperate barnacle *Semibalanus balanoides* (Linnaeus) [26–28]. All specimens were collected alive and matched by developmental stage to minimize ontogenetic variation in skeletal properties [29]. Sample sizes were standardized: *P. violacea* and *S. balanoides* individuals measured ~2 mm in diameter; *H. scutulata* colonies were ~5 mm.

### Sample preparation and elemental analysis

Each specimen was examined under a stereomicroscope to ensure the absence of epibionts (e.g., foraminifera) that could affect mineralogical integrity. Only epibiont-free individuals were selected, with a minimum of five specimens per site to support statistical robustness. Powdered samples (0.003–0.345 g) were weighed using a five-digit analytical balance and transferred to 15 ml Sarstedt® tubes. Samples were digested in 1.5 ml concentrated HNO₃ (Sigma Aldrich® Trace SELECT), 1.5 ml ultrapure water, and 0.3 ml 30% H₂O₂ (Merck® Suprapure), then incubated at 70 °C for 24 hours. Solutions were diluted to 15 ml by weight with ultrapure water.

Elemental analyses were conducted at the Imaging and Analysis Centre, Natural History Museum, London, using a Thermo iCap 6500 Duo ICP-AES. Data were processed with Thermo Scientific iTEVA software. Each batch included blanks (2% HNO₃), six calibration standards (1 ppm), and internal quality controls. Magnesium concentrations were normalized to calcium and expressed as Mg/Ca molar ratios (mmol/mol). Analytical precision and accuracy were verified using two certified reference materials (JLs-1 limestone and JDo-1 dolomite, Geological Survey of Japan), diluted to match sample Ca concentrations. Measured Ca and Mg values were within one standard deviation of certified values [30].

### Statistical analyses

Differences in Mg/Ca ratios among species were tested using non-parametric statistics due to violations of normality (Shapiro-Wilk test) and homogeneity of variance (Levene’s test). Kruskal-Wallis tests were performed for species-level comparisons, followed by Dunn’s post-hoc tests with Bonferroni adjustment to identify pairwise differences.

Interannual variability and temporal trends in bottom-water temperature were examined using high-frequency environmental measurements (every 30 minutes) from in situ loggers deployed in Isfjorden, at the study sites (S1, S2) and depths (6 and 12 m) for the period 2006–2009. Seawater temperature data were averaged by month and quality-controlled by removing missing values prior to analysis.

Linear regression analyses were used to quantify interannual trends in Mg/Ca for each species and to evaluate their relationship with bottom-water temperature. Temporal trends in temperature were calculated by regressing monthly mean values against time expressed as a continuous fractional year, with the slope representing the rate of change (°C year ⁻ ¹) and R² and p-values indicating trend strength and statistical significance.

## Results

### Species specific patterns in skeletal Mg/Ca ratio

**Fig 3:**
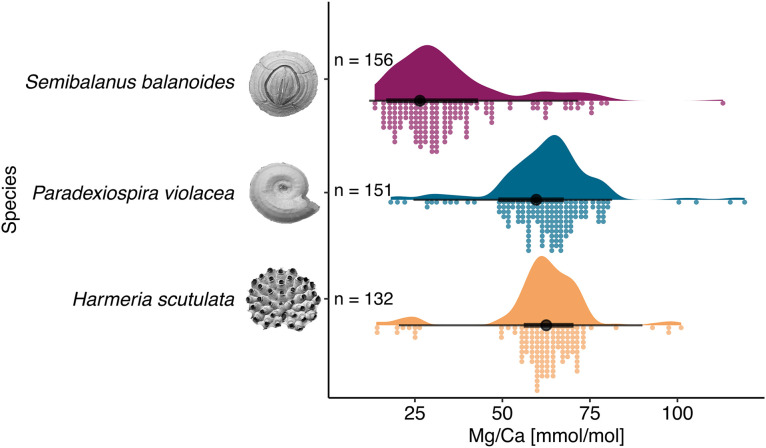
Raincloud plot showing Mg-calcite distribution in three Arctic species: barnacle *S. balanoides*, spirorbis *P. violacea* and bryozoa *H. scutulata.* The violin plot represents the probability density function of observations; the black dot is the sample mean alongside the interquartile range (black line). Dot plots display a histogram-like distribution of individual data observations.

Mg/Ca ratios were measured in 439 specimens across three species (S1 Appendix). Raincloud plots (Fig 3) show clear interspecific differences: *Semibalanus balanoides* had the lowest mean Mg/Ca (35.2 ± 16.8, *n* = 156) and highest variability, while *Paradexiospira violacea* and *Harmeria scutulata* exhibited higher, more consistent ratios (62.5 ± 14.3 and 65.3 ± 14.1, respectively). Kruskal-Wallis test (Kruskal-Wallis χ² = 239.95, df = 2, p < 0.001) reveal that *S. balanoides* had significantly lower Mg/Ca ratios than both *H. scutulata* and *P. violacea* (Dunn’s test, p < 0.001). The difference between *H. scutulata and P.violacea* was marginally non-significant (p = 0.059).

### Spatial variability in skeletal Mg/Ca ratio

**Fig 4:**
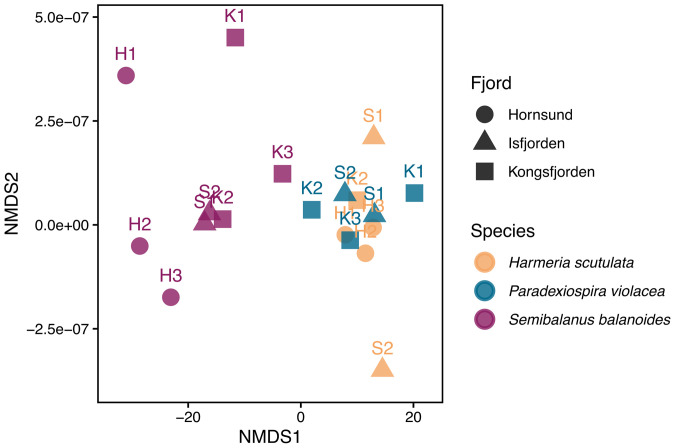
Non-metric multidimensional scaling (NMDS) ordination of skeletal Mg/Ca ratio across species and sampling sites. Each point represents the average Mg/Ca composition for a site, colored by species. Shapes refers to sampling sites, as shown in the legend.

Non-metric multidimensional scaling (NMDS) confirmed strong species-specific separation, with *S. balanoides* occupying a distinct ordination space (Fig 4), whereas site-level patterns were less pronounced, showing substantial overlap. The low stress value of the ordination indicates a good representation of the data in reduced dimensional space.

**Fig 5:**
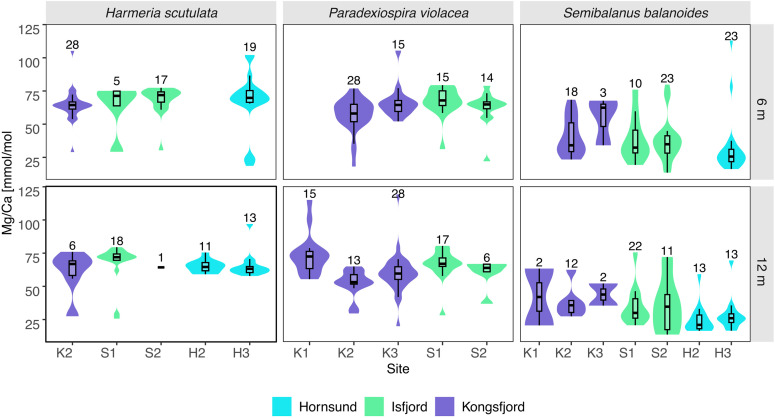
The violin plots showing spatial variability in Mg/Ca of *Harmeria scutulata*, *Paradexiospira violacea*, and *Semibalanus balanoides* between sampling sites (H2, H3, K1, K2, K3, S1, S2) and along depth (6 m, 12 m). Each violin plot illustrates the density distribution of Mg/Ca ratios, with boxplots inside indicating the median and interquartile range (IQR). The sample sizes for each plot are showed above each violin.

Violin plots were used to visualize the distribution and variability of skeletal Mg/Ca ratios across species, sites, and depths, providing a clear representation of data density, spread, and central tendencies within each group (Fig 5). High, within-site, intraspecific variability in Mg/Ca was found in all species but particularly among *P. violacea* and *S. balanoides* (Fig 5). Among the studied taxa, *H. scutulata* exhibited the highest variability at 6 m in Hornsund (H3, SD = 22.5), while *P. violacea* showed broad Mg/Ca ranges at 12 m in Kongsfjorden (K1: SD = 15.8; K3: SD = 16.2). *S. balanoides* displayed the greatest variability in Mg/Ca ratio at 12 m in K1 (SD = 30.3), followed by S2 (SD = 20.5) and H3 (SD = 21.6).

Pairwise Kruskal-Wallis post hoc tests revealed significant site-level differences in Mg/Ca ratios for all species, following a consistent fjord-scale gradient: Hornsund (H) exhibited the lowest values, Kongsfjorden (K) intermediate, and Isfjorden (I) the highest (Table 1). For *S. balanoides*, Mg/Ca ratios were significantly lower at Hornsund (H3) compared to Kongsfjorden (K2) and Isfjorden sites (S1, S2; Z = −5.72 to −4.07, p_adj_ < 0.001). *H. scutulata* also showed lower Mg/Ca at Hornsund (H2, H3) relative to Kongsfjorden and Isfjorden, with significant differences between K2 and S2 (Z = 4.27, p_adj_ = 2.95 × 10 ⁻ ⁴) and within Isfjorden sites S1 and S2 (Z = 2.93, p_adj_ = 0.050). *P. violacea* exhibited significant difference between Kongsfjorden (K2) and Isfjorden (S2; Z = 3.22, p_adj_ = 0.013; Table 1). Depth as a variable did not significantly influence Mg/Ca ratios across species.

**Table 1:** Pairwise comparisons of significant differences in skeletal Mg/Ca ratios among sampling sites within each species using Kruskal-Wallis post hoc tests.

Species	Site comparison	Z	Adjusted p-value
*S. balanoides*	H3 - K2	−5.72	2.95 × 10 ⁻ ⁷
*S. balanoides*	H3 - S1	−4.07	1.34 × 10 ⁻ ³
*S. balanoides*	H3 - S2	−4.17	8.38 × 10 ⁻ ⁴
*P. violacea*	K2 - S2	3.22	0.013
*H. scutulata*	H2 - K2	−4.74	3.25 × 10 ⁻ ⁵
*H. scutulata*	H3 - K2	−5.03	7.36 × 10 ⁻ ⁶
*H. scutulata*	H2 - S1	−3.59	4.92 × 10 ⁻ ³
*H. scutulata*	H3 - S1	−3.27	1.59 × 10 ⁻ ²
*H. scutulata*	K2 - S2	4.27	2.95 × 10 ⁻ ⁴
*H. scutulata*	S1 - S2	2.93	5.03 × 10 ⁻ ²

**Legend: Hornsund (H2, H3), Kongsfjorden (K2), Isfjorden (S1, S2), significant differences are indicated by Z-values, adjusted p-values.**

### Temporal variability in Mg/Ca ratio between species

**Fig 6:**
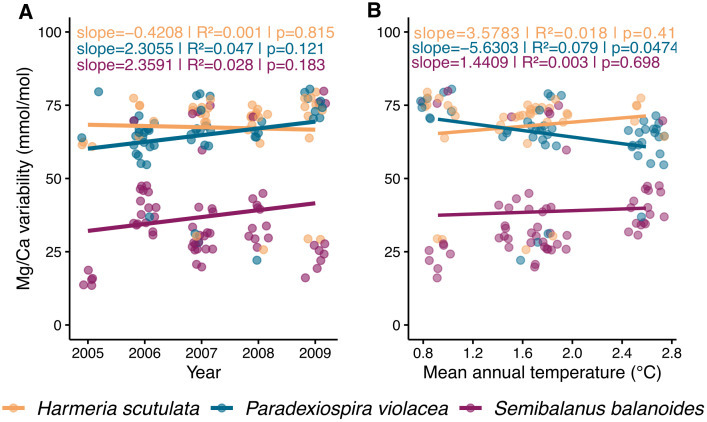
Temporal variability in Mg/Ca ratios (mmol/mol) from 2005–2009 for three marine calcifiers *H. scutulata*, *P. violacea* and *S. balanoides*, at Isfjorden. A) Average Mg/Ca ratio versus year per species. Colored points and trend lines represent different **species, as described in the legend. B) Mean annual Mg/Ca versus mean annual sea water temperature per species. Regression lines (linear models) and corresponding statistics (slope, R², p-value) are shown in the top-left corner of each panel.**

Linear regression analysis revealed a positive but mostly non-significant trends in Mg/Ca between 2006 and 2009 for all species. *S. balanoides* showed a positive slope of 2.359 (R^2^ = 0.028, p = 0.183), *P. violacea* had a slope of 2.305 (R^2^ = 0.047, p = 0.121), and *H. scutulata* showed a slight negative trend (slope = −0.421, R^2^ = 0.001, p = 0.815; Fig 6A). Overall non of these trends were statistically significant, indicating weak temporal change in Mg/Ca across the study period.

Regression between Mg/Ca and mean annual temperature showed weak relationships, with only *P. violacea* displaying a marginally significant negative trend (slope = −5.630, R^2^ = 0.079, p = 0.047). For *S. balanoides*, the slope was −1.441 (R^2^ = 0.003, p = 0.698), and for *H. scutulata*, the slope was 3.578 (R^2^ = 0.018, p = 0.410; Fig 6B). Thus, Mg/Ca appears largely independent from short-term temperature variation in Isfjorden.

**Fig 7:**
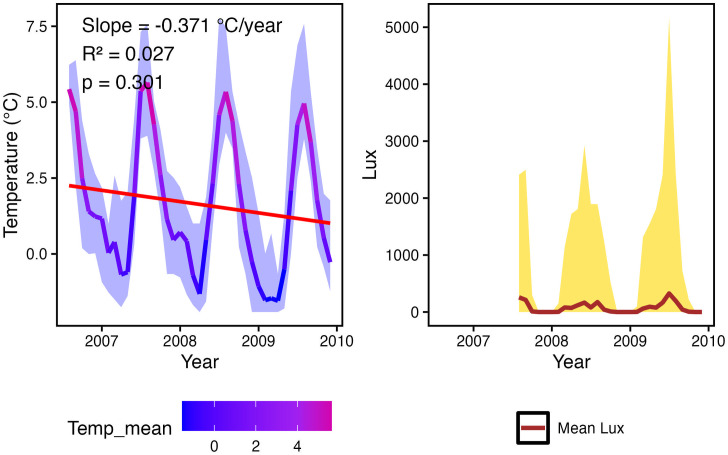
Monthly-averaged temperature and light (Lux) variability in Isfjorden between 2006 and 2009. Left panel: Monthly mean temperature (colored line) with minimum and maximum monthly values shown as a shaded blue ribbon. A trend line (red) is included, with slope, R², and p-value displayed in the top-left corner. Right panel: Monthly mean light levels (Lux; brown line) with minimum and maximum monthly values indicated by a shaded yellow ribbon.

Between 2006 and 2009, mean annual temperature exhibited a decreasing trend, with an average rate of decline of 0.371 °C yr ⁻ ¹. This trend, however, was not statistically significant (p < 0.3) and explained only a small fraction of the observed variability (R² = 0.027; Fig 7).

## Discussion

### Species specific patterns in skeletal Mg/Ca ratio

Our results reveal pronounced species-specific differences in skeletal Mg/Ca ratios (Figs 3-5). Kruskal-Wallis analysis indicates that *Semibalanus balanoides* has significantly lower Mg/Ca ratios (<50 mmol/mol; mean 35.2 ± 16.8 mmol/mol) and the highest intraspecific variability compared with *Paradexiospira violacea* and *Harmeria scutulata* (Table 1; Figs 3-5; S1 Appendix, S2 Appendix). Overall, our results align with global trends in barnacle geochemistry, showing that these sessile invertebrates typically secrete low-Mg calcite (generally <50 mmol/mol) while maintaining elevated Sr/Ca ratios averaging ~4.2 mmol/mol [31] deviating from the typical Sr–Mg correlations seen in inorganic carbonates [32]. The observed, low-Mg concentration in *S. balanoides* and barnacles in general, is most likely result of conservative biomineralization strategy of thoracican barnacles. This group evolved low-Mg calcite shells in the Jurassic and have maintained this trait until today, despite extensive ecological diversification [31]. Thoracican barnacles biomineralize extracellularly within a mantle-derived, cell-lined compartments, where ion transport and pH regulation enable controlled precipitation of Mg-calcite [31,33–37]. Intra-individual variability in Mg/Ca observed in our results (Figs 3-5) may reflect anatomical differentiation, as Mg concentrations in scuta and terga are approximately 40% higher than in wall plates [38]. Although such variability among barnacles has been documented so far only within Balaniformes, similar patterns occurr in echinoderms [7,38–41], brachiopods [42] and bivalves [43]. The conservative and specific/unique biomineralization strategy of thoracican barnacles, particularly lead to consistently lower Mg/Ca ratio in comparison with the *H. scutulata* and *P. violacea*, representing distinct phylogenetic lineages. Similar differences in Mg/Ca ratios have been reported elsewhere, e.g., between the baltic barnacle *Amphibalanus improvisus* and the bryozoan *Einchorinia crustulenta* confirming species-specific responses to the same environmental conditions [44]. In contrast, *P. violacea* and *H. scutulata* exhibit higher and more consistent Mg/Ca ratios (62.5 ± 14.3 and 65.3 ± 14.1 mmol/mol, respectively; Figs 3-5), suggesting stronger biological regulation of Mg incorporation. In both taxa, mineral precipitation occurs onto organic matrices secreted by epithelial tissues, providing controlled nucleation surfaces and facilitating selective incorporation of Mg²⁺ and other trace elements [34,45–49]. Thus the site and mechanism of mineral formation strongly influence on the element/Ca, what could also cause the differences between taxaonomic groups in our study. The intracellular mechanism allow for more precise regulation of pH and ion concentrations and stronger control over Mg/Ca ratios but it is common for as taxa like coccolithophores, foraminifera, and corals [50–52,53]. In contrast species such as bryozoans, serpulids, barnacles or molluscs, regulate Mg² ⁺ incorporation through ion transport, vacuolization, and organic matrix templating [45–49,54,53], allowing species-specific but less controlled Mg incorporation. Both serpulids and bryozoans, in their evolution history, have undergone similar – repeated transitions between low and high-Mg calcite, and in some cases aragonite, and these shifts are constrained by lineage-specific biomineralization mechanisms [1,45–49,55–57]. Although mineralogical transitions might reflect adaptive responses to environmental gradients, particularly those related to temperature and latitude, their expression remains strongly influenced by evolutionary history [1,31,55–59]. Consequently, described similarities in biomineralization strategy and evolution history lead, most likely to similar Mg/Ca levels between spirorbids and bryozoans but distinct to *S. balanoides.* Similar results were found by Ullrich et al. [60]. This study based on 18 benthic marine invertebrate species spanning multiple carbonate polymorphs (low-Mg, high-Mg calcite, aragonite or mixed mineralogy) and various phyla (Mollusca, Echinodermata, Arthtopoda, Annelida, Cnidaria, Chlorophyta and Rhodophyta), showed strong species-specific levels of elemental ratios, with phylogeny explaining more variance than mineralogy for most elements [60]. Species-level patterns found in our study align with this broad comparative study showing that elemental ratios, including Mg/Ca, in biogenic carbonates are primarily structured by phylogeny and biomineralization strategy and outweigh external drivers such as, e.g., seawater chemistry [29,59,60]. Although Mg incorporation may still vary with age, growth rate, or reproductive condition or might be species-specific reaction to external environmental conditions [29,32,61].

### Spatial variability in skeletal Mg/Ca ratio

Sampling location was found as the second most important factor influencing skeletal Mg/Ca ratios across the three studied taxa, as indicated by non-metric multidimensional scaling (NMDS; Fig 4) and supported by Kruskal-Wallis pairwise comparisons (Table 1). We found a spatial pattern in Mg/Ca ratio across all taxa: H (Hornsund) < I (Isfjorden) < K (Kongsfjorden). For instance, in *S. balanoides* as in *H. scutulata*, consistently lower Mg/Ca was observed at Hornsund sites (H3 and H2) in comparison with K2 or S1, S2 (Fig 5, Table 1). While a pattern among locations of K2 > S2 in Mg/Ca ratio was found in both *H. scutulata* (Z = 4.27, p_adj_ = 2.95 × 10 ⁻ ⁴) and *P. violacea* (Z = 3.22, p_adj_ = 0.013). Within-fjord differences were recorded exclusively in *H. scutulata* between the Isfjorden sites (S1–S2: Z = 2.93, p_adj_ = 0.0503), suggesting that fjord-scale environmental gradients are the dominant driver of Mg/Ca variability, although possibly modulated by microhabitat-specific factors such as, e.g., temperature fluctuations, freshwater input, sedimentation or presence/absence of kelp forests. Similar patterns have been reported in Antarctic bryozoans and spirorbids [62–63]. The three fjords studied form a clear temperature–salinity gradient. Hornsund fjord (H-sites) is the coldest (surface <3 °C, 33.3–34.7 PSU) with high nutrient input from glacial meltwater and seabirds, supporting high turbidity and primary production (~216 g C m ⁻ ² y ⁻ ¹) dominated by microplankton [11–17,23]. Isfjorden (I-sites) has intermediate conditions (≈4.9 °C, > 34.7 PSU), reflecting a mix of Arctic and Atlantic waters [15,19–21]. Kongsfjorden (K-sites) is the warmest (~1 °C above Hornsund, ~ 0.5 PSU more saline) but experiences episodic freshening (<28 PSU), lower primary production (~48 g C m ⁻ ² y ⁻ ¹), and reduced carbonate saturation [15–20,22,23]. Warmer and more saline waters generally enhance Mg incorporation in marine calcifiers [64,65,66], which could also explains the observed H < I < K pattern in our study. Barnacles can show large variability in skeletal Mg/Ca even where seasonal temperatures are relatively stable [31], while across broad geographic gradients these organisms can still exhibit higher Mg/Ca in warmer tropical waters and lower values in polar regions [31,37,67,68]. Temperature-related trends have been reported in coralline algae, brachiopods, foraminifera, echinoderms, and bryzoans although the strength of the relationship often varies among species [ 29,56–59,65,69–73]. Thus, while the H < I < K pattern suggests a temperature influence on skeletal Mg/Ca, the substantial intra-specific and within-site variability observed in our results indicates that Mg/Ca incorporation cannot be explained as solely temperature-driven.

Consistent with these fjord-scale gradients, seawater chemistry can also vary substantially within a single fjord [74–76], depending on proximity to glaciers and river inputs as well as the presence of kelp forests which can create its own specific environment [ 20,23,77–81]. Sites influenced by glacial meltwater (particularly in Hornsund) or river plumes (e.g., S1 and S2) therefore often experience locally reduced salinity and alkalinity, resulting in lower pH and carbonate saturation (Ω) which potentially can lead to differences in skeletal Mg/Ca among individuals from different locations.

Indeed reduced ionic competition under lower salinity can modify Mg uptake and alter Mg/Ca ratios in calcitic skeletons, as documented in barnacles, bryozoans, molluscs, and coralline algae [20,23,65,81–84]. In our study, lower skeletal Mg/Ca ratios observed at Hornsund sites are therefore consistent with the strong glacial freshwater influence and reduced salinity characteristic of this fjord. However, the lack of a uniform response among our species confirms that salinity effects on Mg incorporation are taxon-specific, and closely related species may respond differently. For example, along the strong salinity gradient of the Baltic Sea, contrasting respons was found among barnacles of *Amphibalanus improvisus* which exhibits lower Mg/Ca in lower salinity, whereas *S. balanoides* shows the opposite pattern under comparable conditions. Positive increase in Mg/Ca with salinity have been reported for the bryozoan *Cryptosula pallasiana* and several spirorbid polychaetes [82]. Low Ω may further constrain Mg uptake and favour precipitation of low-Mg calcite, particularly in Mg-rich taxa such as serpulids and coralline algae, which may form brittle or compositionally altered skeletons [66,67]. Barnacles appear to be influenced by changes in carbonate saturation mainly through calcification and growth, with limited direct effects on Mg incorporation, and bivalves generally show stable Mg/Ca unless conditions change rapidly [66,69]. The presence of protective organic layers, such as periostraca or epicuticles, may further reduce dissolution risk, although the effectiveness of these barriers varies among taxa [7,70].

In contrast, presence of kelp forests can partially buffer unfavourable conditions in seawater chemistry through photosynthetic uptake of CO₂ and HCO₃ ⁻ , locally elevating pH and Ω [77–80]. In our study, structurally complex kelp forests were found at sites, e.g., S1, S2 and K2 dominated by Saccharina *latissima*, Laminaria digitata or Alaria esculenta [78,79]. Lower seawater pH (e.g., outside kelp forests) may affect ability to regulate calcifying fluid chemistry by the species. While lower pH generally reduces overall shell weight and calcification rates, its effect on Mg/Ca ratios, also varies depending on the organism. The energetic cost of maintaining skeletal integrity differs between high-Mg (e.g., coralline algae, echinoderms, bryozoans, serpulids) and low-Mg calcifiers (e.g., foraminifera). Generally, taxa that exert stronger biological control (e.g., foraminifera) show less variation in Mg/Ca [50,85].

The contrasting responses to both microhabitat and fjord-scale environmental gradients among co-occurring taxa further suggest that Mg/Ca ratios are primarily governed by species-specific factors, such as growth, metabolism, and biomineralization pathways, rather than being solely driven by environmental variables, e.g., temperature or salinity [9,59,60,86].

### Temporal variability in Mg/Ca ratio between species

Interannual analyses of skeletal Mg/Ca ratios in Isfjorden revealed species-specific temporal patterns (Fig 6A). While *P. violacea* and *S. balanoides* displayed positive temporal slopes, in contrast to *H. scutulata*, none of the recorded trends were statistically significant. Linear regressions between Mg/Ca and contemporaneous bottom-water temperatures revealed weak and mostly non-significant relationships, with only *P. violacea* showing a marginally significant negative correlation (Fig 6B). Between 2006 and 2009, mean annual bottom temperatures at our study sites, decreased from ~2.5°C to ~1°C; however, this represents rather a temporary fluctuation within a longer-term warming trajectory. Previous observations indicate a clear warming trend in Isfjorden of ~1°C per decade over the past 30 years [21], accompanied by longer sea-ice-free periods (2000–2023) and rising bottom temperatures of ~0.5°C per decade between 2006 and 2022, particularly at our study sites [87].

Temporal Mg/Ca variability has previously been documented in other Antarctic bryozoans and spirorbids, but consistent and statistically significant trends were lacking [62,73]. Similarly, a 30-year study of Antarctic bryozoan species *Cellaria diversa* (Livingstone, 1928) and *Antarcticaetos bubeccata* (Rogick, 1955) revealed no significant differences in skeletal Mg-calcite [73]. Although experimental and global-scale studies demonstrate temperature-dependent Mg incorporation [31,49,59], such signals may be masked in polar regions by low seasonal variability, local environmental fluctuations, and species-specific controls.

These findings suggest that Mg/Ca responses in polar calcifiers may be less predictable over short timescales than, for instance, in temperate systems [82]. They also indicate that Mg/Ca is not always strongly temperature-dependent, particularly in regions when temperature variability is small and within a narrow annual range. In such cases, Mg/Ca may reflect the integrated influence of multiple environmental drivers such as seawater biogeochemistry, salinity, food availability, but also physiological regulations, including metabolic activity, growth rate, and ontogeny, which can override direct thermal control. Overall, the species-specific temporal trends and the weak temperature dependence suggest that Mg/Ca variability in these species is not strongly driven by short-term thermal changes. Instead, intrinsic biological regulation and interannual variability in growth and calcification processes likely play a central role. Thus, our hypothesis that inter annual Mg/Ca changes are directly coupled to temperature is not supported for the 2006–2009 interval. Longer time series and higher-resolution temperature records would be needed to determine whether Mg/Ca tracks climatic warming over decadal scale.

## Conclusions

Our study demonstrates that skeletal Mg/Ca ratios in Arctic calcifying invertebrates are primarily controlled by species-specific physiology reflecting fundamental differences in phylogeny and biomineralization mechanisms. Fjord-scale differences in temperature, salinity, and freshwater input shape overall Mg/Ca spatial patterns (Hornsund < Isfjorden < Kongsfjorden), while local microhabitat factors, such as carbonate chemistry, food availability, sedimentation, and kelp canopy, likely drive site-specific and intra-specific variability. Temporal analysis in Isfjorden revealed species-specific interannual variability in Mg/Ca with for *Harmeria scutulata* and *Semibalanus balanoides*, whereas *Paradexiospira violacea* remained stable, indicating that Mg incorporation can change over short timescales independently of bottom-water temperature. These patterns highlight how intrinsic biological processes interact with environmental variability to produce complex geochemical signals. Overall, our findings emphasize the need for taxon-specific, long-term monitoring that integrates geochemical, ecological, and oceanographic data to interpret Mg/Ca proxies accurately and evaluate the resilience of Arctic calcifiers under ongoing warming and freshening.

## Supporting information

S1 Appendix Geochemical and sampling data for the three studied species *Harmeria scutulata, Paradexiospira violacea,* and *Semibalanus balanoides.* (XLSX)

S2 Appendix Summary statistics for skeletal Mg/Ca ratios and species, site/depth and year displayed in the main-text figures. Each table presents the mean, standard deviation (SD), minimum (min), maximum (max), and sample size (n).
(DOCX)
