# Supplementary material for: Intrinsic over extrinsic: Species identity shapes spatial and interannual Mg/Ca patterns in Arctic marine calcifiers
Source: PLoS One. 2026 Mar 20;21(3):e0345703. doi: 10.1371/journal.pone.0345703 (PMC13004348; doi:10.1371/journal.pone.0345703)
Supplement: S2 Appendix — Each table presents the mean, standard deviation (SD), minimum (min), maximum (max), and sample size (n). (DOCX) [file pone.0345703.s002.docx]

**Table 1.** Summary statistics of skeletal Mg/Ca ratios by species. For each species, the table presents the sample size (N), mean Mg/Ca ratio, standard deviation (SD), and the minimum (min) and maximum (max) observed values. These descriptive statistics provide the basis for interspecific comparisons presented in the Fig. 3

| **Species** | **N** | **Mean Mg/Ca [mmol/mol]** | **SD** | **Min Mg/Ca** | **Max Mg/Ca** |
| --- | --- | --- | --- | --- | --- |
| ***Harmeria scutulata*** | **132** | **65.34** | **14.1** | **18.1** | **105.2** |
| ***Paradexiospira violacea*** | **151** | **62.47** | **14.29** | **18.1** | **119.2** |
| ***Semibalanus balanoides*** | **156** | **35.17** | **16.82** | **13.5** | **113** |

**Table 2.** Summary statistics of skeletal Mg/Ca ratios by species, sampling site, and depth. For each group, the table presents sampling depth (m), sample size (N), mean Mg/Ca ratio (mmol/mol), standard deviation (SD), and the minimum (min) and maximum (max) observed values. These statistics support comparisons among species and across sites and depth gradients, presented on Fig. 5

| **Species** | **Site** | **Depth [m]** | **N** | **Mean Mg/Ca [mmol/mol]** | **SD**  **Mg/Ca** | **Min Mg/Ca** | **Max Mg/Ca** |
| --- | --- | --- | --- | --- | --- | --- | --- |
| *Harmeria scutulata* | H3 | 6 | 19 | 66.96 | 22.54 | 18.6 | 102.1 |
|  | S1 | 6 | 5 | 62.86 | 19.36 | 29.2 | 75.1 |
|  | K2 | 6 | 28 | 64.32 | 11.78 | 29 | 105.2 |
|  | S2 | 6 | 17 | 68.35 | 11.11 | 30.3 | 77.4 |
|  | K2 | 12 | 6 | 60.42 | 17.42 | 27.5 | 76 |
|  | S1 | 12 | 18 | 67.61 | 15.08 | 25.7 | 79.5 |
|  | H3 | 12 | 13 | 65.74 | 10.02 | 57.8 | 97 |
|  | H2 | 12 | 11 | 65.11 | 4.87 | 59.1 | 75.5 |
|  | S2 | 12 | 1 | 64.3 | NA | 64.3 | 64.3 |
| *Paradexiospira violacea* | S2 | 6 | 14 | 62.14 | 13.13 | 22.1 | 78.9 |
|  | K2 | 6 | 28 | 57.14 | 12.91 | 18.1 | 76.9 |
|  | K3 | 6 | 15 | 66.52 | 12.34 | 52.1 | 105.4 |
|  | S1 | 6 | 15 | 67.02 | 11.82 | 31.2 | 79.4 |
|  | K3 | 12 | 28 | 60.19 | 16.15 | 20 | 119.2 |
|  | K1 | 12 | 15 | 73.81 | 15.85 | 55.4 | 115.1 |
|  | S1 | 12 | 17 | 66.44 | 11.67 | 28.2 | 80.5 |
|  | S2 | 12 | 6 | 59.87 | 11.58 | 36.9 | 66.9 |
|  | K2 | 12 | 13 | 52.19 | 10.37 | 29.6 | 65 |
| *Semibalanus balanoides* | H3 | 6 | 23 | 31.60 | 21.64 | 16.2 | 113 |
|  | K3 | 6 | 3 | 54.73 | 18.06 | 34.1 | 67.7 |
|  | S1 | 6 | 10 | 39.06 | 17.41 | 19.3 | 76 |
|  | S2 | 6 | 23 | 38.1 | 16.70 | 13.5 | 79.8 |
|  | K2 | 6 | 18 | 40.74 | 14.24 | 23.4 | 68.4 |
|  | K1 | 12 | 2 | 42.00 | 30.26 | 20.6 | 63.4 |
|  | S2 | 12 | 11 | 36.01 | 20.53 | 13.7 | 72.1 |
|  | S1 | 12 | 22 | 35.43 | 14.99 | 20.7 | 75.6 |
|  | H3 | 12 | 13 | 28.71 | 13.30 | 16.6 | 69.5 |
|  | K2 | 12 | 12 | 38.26 | 11.92 | 27.4 | 62.5 |
|  | K3 | 12 | 2 | 43.85 | 11.81 | 35.5 | 52.2 |
|  | H2 | 12 | 13 | 25.04 | 11.51 | 16.5 | 59.2 |

**Table 3.** Annual Mg/Ca ratios for each species in Isfjord. For each year, the mean Mg/Ca ratio [mmol/mol] is presented along with the standard deviation (SD), minimum and maximum values, and the total number of measurements (N). Values summarize all sites and depths sampled per species.

| Species | **Year** | **Mean Mg/Ca [mmol/mol]** | **SD Mg/Ca** | **Min Mg/Ca** | **Max Mg/Ca** | **N** |
| --- | --- | --- | --- | --- | --- | --- |
| *Harmeria scutulata* | 2005 | 61.600 | 0.755 | 60.9 | 62.4 | 3 |
|  | 2006 | 72.200 | 5.276 | 64.3 | 77.4 | 5 |
|  | 2007 | 68.540 | 13.646 | 30.3 | 77.0 | 10 |
|  | 2008 | 66.700 | 13.365 | 25.7 | 75.1 | 12 |
|  | 2009 | 65.991 | 18.598 | 29.2 | 79.5 | 11 |
| *Paradexiospira violacea* | 2005 | 71.750 | 11.102 | 63.9 | 79.6 | 2 |
|  | 2006 | 62.285 | 7.557 | 36.9 | 71.4 | 20 |
|  | 2007 | 63.831 | 14.481 | 28.2 | 78.9 | 16 |
|  | 2008 | 58.617 | 17.994 | 22.1 | 69.4 | 6 |
|  | 2009 | 75.225 | 3.801 | 70.4 | 80.5 | 8 |
| *Semibalanus balanoides* | 2005 | 15.460 | 2.100 | 13.5 | 18.7 | 5 |
|  | 2006 | 41.176 | 9.245 | 30.7 | 69.7 | 17 |
|  | 2007 | 36.805 | 16.981 | 19.8 | 74.9 | 22 |
|  | 2008 | 38.508 | 11.875 | 26.5 | 71.0 | 12 |
|  | 2009 | 39.330 | 26.341 | 16.1 | 79.8 | 10 |

**Table 4.** Monthly summary statistics of bottom-water temperature (°C) and light intensity (lux) recorded by HOBO data loggers at Isfjorden. Temperature and lux were measured at 30-minute intervals and subsequently averaged to obtain monthly mean values from two sites (S1, S2) and depths (6 and 12 m). For each month, the table presents the mean, minimum (min), and maximum (max) values.

| **Month_year** | **Temp_mean** | **Temp_min** | **Temp_max** | **Lux_mean** | **Lux_min** | **Lux_max** |
| --- | --- | --- | --- | --- | --- | --- |
| 1.08.2006 | 5.430 | 4.973 | 6.230 | NA | NA | NA |
| 1.09.2006 | 4.700 | 2.262 | 6.382 | NA | NA | NA |
| 1.10.2006 | 2.491 | 0.19 | 4.402 | NA | NA | NA |
| 1.11.2006 | 1.402 | -0.004 | 3.301 | NA | NA | NA |
| 1.12.2006 | 1.235 | 0.024 | 2.637 | NA | NA | NA |
| 1.01.2007 | 1.165 | -0.93 | 2.182 | NA | NA | NA |
| 1.02.2007 | 0.035 | -1.27 | 0.989 | NA | NA | NA |
| 1.03.2007 | 0.394 | -1.498 | 2.584 | NA | NA | NA |
| 1.04.2007 | -0.681 | -1.756 | 1.751 | NA | NA | NA |
| 1.05.2007 | -0.600 | -1.384 | 2.047 | NA | NA | NA |
| 1.06.2007 | 1.915 | 0.301 | 4.506 | NA | NA | NA |
| 1.07.2007 | 5.368 | 3.801 | 7.318 | NA | NA | NA |
| 1.08.2007 | 5.649 | 3.893 | 7.569 | 257.996 | 0 | 2411.1 |
| 1.09.2007 | 4.265 | 2.73 | 5.037 | 212.826 | 0 | 2497.2 |
| 1.10.2007 | 2.603 | 1.73 | 4.102 | 9.949 | 0 | 301.4 |
| 1.11.2007 | 1.133 | -0.662 | 2.731 | 0 | 0 | 0 |
| 1.12.2007 | 0.462 | -0.662 | 2.517 | 0 | 0 | 0 |
| 1.01.2008 | 0.713 | -0.774 | 2.195 | 0 | 0 | 0 |
| 1.02.2008 | 0.417 | -1.341 | 1.548 | 2.482 | 0 | 150.7 |
| 1.03.2008 | -0.703 | -1.684 | 1.003 | 79.734 | 0 | 1141 |
| 1.04.2008 | -1.318 | -1.914 | 1.003 | 72.034 | 0 | 1722.2 |
| 1.05.2008 | 0.464 | -1.57 | 1.872 | 119.612 | 0 | 1808.3 |
| 1.06.2008 | 2.323 | 0.893 | 4.102 | 165.440 | 0 | 2927.8 |
| 1.07.2008 | 4.577 | 2.943 | 7.782 | 79.803 | 0 | 1894.5 |
| 1.08.2008 | 5.342 | 3.998 | 7.481 | 174.547 | 0 | 1894.5 |
| 1.09.2008 | 4.380 | 3.472 | 5.347 | 41.884 | 0 | 1237.9 |
| 1.10.2008 | 2.310 | 0.232 | 4.311 | 9.361 | 0 | 516.7 |
| 1.11.2008 | 0.779 | -0.662 | 3.367 | 0 | 0 | 0 |
| 1.12.2008 | -0.261 | -1.914 | 2.517 | 0 | 0 | 0 |
| 1.01.2009 | -1.069 | -1.914 | 1.33 | 0 | 0 | 0 |
| 1.02.2009 | -1.524 | -1.914 | 0.01 | 1.626 | 0 | 96.9 |
| 1.03.2009 | -1.463 | -1.914 | 0.674 | 60.335 | 0 | 1313.2 |
| 1.04.2009 | -1.543 | -1.914 | -0.662 | 92.793 | 0 | 1550 |
| 1.05.2009 | -0.506 | -1.799 | 1.33 | 77.195 | 0 | 1808.3 |
| 1.06.2009 | 2.090 | 0.01 | 5.347 | 166.034 | 0 | 2411.1 |
| 1.07.2009 | 4.264 | 2.517 | 6.877 | 325.992 | 0 | 5166.7 |
| 1.08.2009 | 4.959 | 3.788 | 7.582 | 195.481 | 0 | 2411.1 |
| 1.09.2009 | 3.684 | 2.41 | 5.347 | 43.111 | 0 | 721.2 |
| 1.10.2009 | 1.768 | 0.232 | 3.472 | 4.824 | 0 | 215.3 |
| 1.11.2009 | 0.533 | -0.437 | 1.98 | 0 | 0 | 0 |
| 1.12.2009 | -0.273 | -1.228 | 1.764 | 0 | 0 | 0 |
